# Response of Physiological, Reproductive Function and Yield Traits in Cultivated Chickpea (*Cicer arietinum* L.) Under Heat Stress

**DOI:** 10.3389/fpls.2022.880519

**Published:** 2022-05-25

**Authors:** Poonam Devi, Uday Chand Jha, Vijay Prakash, Sanjeev Kumar, Swarup Kumar Parida, Pronob J. Paul, P. V. Vara Prasad, Kamal Dev Sharma, Kadambot H. M. Siddique, Harsh Nayyar

**Affiliations:** ^1^Department of Botany, Panjab University, Chandigarh, India; ^2^ICAR–Indian Institute of Pulses Research, Kanpur, India; ^3^Agricultural Research Station (S.K.R.A.U, Bikaner), Sri Ganganagar, India; ^4^Department of Plant Sciences, Central University of Punjab, Bhatinda, India; ^5^National Institute of Plant Genome Research, New Delhi, India; ^6^International Rice Research Institute South-Asia Hub, Hyderabad, India; ^7^Sustainable Intensification Innovation Lab, Kansas State University, Manhattan, KS, United States; ^8^Department of Agricultural Biotechnology, CSK Himachal Pradesh Agricultural University, Palampur, India; ^9^The UWA Institute of Agriculture, The University of Western Australia, Perth, WA, Australia

**Keywords:** chickpea, heat stress, climate resilience, genotype, physiological trait

## Abstract

Under global climate change, high-temperature stress is becoming a major threat to crop yields, adversely affecting plant growth, and ultimately resulting in significant yield losses in various crops, including chickpea. Thus, identifying crop genotypes with increased heat stress (HS) tolerance is becoming a priority for chickpea research. Here, we assessed the response of seven physiological traits and four yield and yield-related traits in 39 chickpea genotypes grown in normal-sown and late-sown environments [to expose plants to HS (>32/20°C) at the reproductive stage] for two consecutive years (2017–2018 and 2018–2019). Significant genetic variability for the tested traits occurred under normal and HS conditions in both years. Based on the tested physiological parameters and yield-related traits, GNG2171, GNG1969, GNG1488, PantG186, CSJ515, RSG888, RSG945, RVG202, and GNG469 were identified as promising genotypes under HS. Further, ten heat-tolerant and ten heat-sensitive lines from the set of 39 genotypes were validated for their heat tolerance (32/20°C from flowering to maturity) in a controlled environment of a growth chamber. Of the ten heat-tolerant genotypes, GNG1969, GNG1488, PantG186, RSG888, CSJ315, and GNG1499 exhibited high heat tolerance evidenced by small reductions in pollen viability, pollen germination, and pod set %, high seed yield plant^–1^ and less damage to membranes, photosynthetic ability, leaf water status, and oxidative processes. In growth chamber, chlorophyll, photosynthetic efficiency, pollen germination, and pollen viability correlated strongly with yield traits. Thus, GNG1969, GNG1488, PantG186, RSG888, CSJ315, and GNG1499 genotypes could be used as candidate donors for transferring heat tolerance traits to high-yielding heat-sensitive varieties to develop heat-resilient chickpea cultivars.

## Introduction

Plants have an optimum temperature range where they perform best based on their genetic makeup; exposure to temperatures beyond the threshold is considered heat stress (HS; Hatfield and Prueger, 2015). Extreme temperature adversely affects crop growth by damaging morphological, physiological, biochemical, and molecular characteristics and ultimately reducing yield (Hemantaranjan et al., 2014; Sehgal et al., 2018; Jameel et al., 2021). At the sub-cellular level, HS impairs vital processes such as photosynthesis, respiration, membrane functioning, and water relations, affecting the functioning of enzymes, proteins, hormones, and primary and secondary metabolites (Bita and Gerats, 2013; Chaudhary et al., 2020). Additionally, HS can induce the accumulation of reactive oxygen species, cause organelles to malfunction, alter phytohormone production and signaling, and induce transcriptomic re-programming and metabolomic changes (Hasanuzzaman et al., 2013). Heat stress is frequently associated with reduced water availability; thus, crops grown in tropical and sub-tropical environments should be evaluated for their HS response (Barnabás et al., 2008; Krishnamurthy et al., 2011).

Temperatures are rising globally due to climate change and anthropogenic reasons (Mukherjee et al., 2018), posing a serious threat to various agricultural crops, including cool-season legumes such as chickpea. Heat stress at critical stages during plant development, especially the reproductive stage, severely constrains chickpea production. Chickpea performs well when the reproductive stage coincides with average temperatures of 20–28°C (Devasirvatham et al., 2013). However, temperatures (>32°C) during flowering and pod filling lead to various anomalies in reproductive organs resulting in flower drop, pollen sterility, pod abortion, and substantial losses in seed yield (Singh et al., 2016; Gaur et al., 2019; Pareek et al., 2019; Jha et al., 2021). Therefore, it is vital to develop heat tolerance in chickpea under the prevailing HS conditions.

Substantial reductions in chickpea yield have been observed for even a 1°C rise in temperature beyond the threshold (Kalra et al., 2008). Canci and Toker (2009) reported large-scale genetic variation in a field evaluation of 377 chickpea germplasm lines and 68 accessions of wild *Cicer* species, identifying several heat-tolerant genotypes and suggesting that harvest index, seed yield, and pods plant^–1^ be considered as selection traits. Similarly, Gaur et al. (2010) studied 180 chickpea genotypes at two locations in India and found large genetic variation for pod number. Information about genotypic diversity in terms of specific morpho-physiological and reproductive traits in chickpea is rarely available, hence the existing chickpea germplasm need to be assessed to identify heat tolerant genotypes and the underlying mechanisms. This study developed an effective, simple, and reliable screening method with well-defined traits for selecting heat-tolerant chickpea genotypes under field conditions. The screening method could be used to identify germplasm with increased heat tolerance for introgression into commercial chickpea breeding programs.

## Materials and Methods

### Field and Growth Chamber Experiments

The seeds of 39 chickpea (*Cicer arietinum* L.) genotypes (Supplementary Table 1) were procured from various sources (Punjab Agricultural University, Ludhiana, India; Indian Institute of Pulse Research, Kanpur, India; Agricultural Research Station Sriganganagar, Rajasthan, India). Genotype ICC92944 (Source: ICRISAT) was used as a heat-tolerant check. Chickpea seeds were sown in pots (8 L capacity) containing a mixture of air-dried soil, sand, and farmyard manure [2:1:1 (v/v)]. The loam soil (pH 7.1) contained 54, 43, and 158 kg ha^–1^ of available N, P, and K, respectively. The seeds were inoculated with *Mesorhizobium* sp. at the recommended rate of 1.95 g kg^–1^ seeds. Five seeds were planted in each pot and thinned to three per pot after emergence (10 pots genotype^–1^ in triplicate). The experiment had a randomized complete block design. Meteorological information (mainly daily temperature and relative humidity profiles) from sowing date to maturity was recorded throughout the cropping season. The plants were grown in a natural environment outdoors in a wire-covered dome to protect them from birds and small animals at Panjab University, Chandigarh, India.

The chickpea genotypes were sown in pots on two sowing dates: (1) first week of November for normal sowing and (2) first week of January for late sowing to impose HS at the reproductive stage following CRBD. The year-wise weather details (Temperature, relative humidity, and light intensity) are given in Supplementary Figure 1 and Supplementary Table 2.

In a separate experiment, conducted in controlled environment in a growth chamber (CRBD), plants of some selected genotypes (10 heat-tolerant, 10 heat-sensitive) at the onset of the bud stage were initially exposed to 25/15°C for a day, followed by a gradual increase by 2°C d^–1^ to obtain the required temperature (32/22°C). The plants were fully irrigated with (water applied twice daily at 10.00 a.m. and 7.00 p.m.) to avoid any drought stress during the heat treatment. The chamber had a light intensity of about 500 μmol m^–2^ s^–1^ and RH of 65–70% during the experiment.

Fresh leaves located at 2nd and 3rd branches from the top in control and heat-stressed plants (15 day after exposure to stress) were assessed for seven physiological traits [Electrolyte leakage (EL); leaf chlorophyll (CHL) content, stomatal conductance (gS), Fv/Fm photosystem II efficiency (PSII), malondialdehyde concentration (MDA)]. At the same time, the flowers (2 days before anthesis) were collected to measure pollen germination % (PGP), pollen viability % (PVP). The plants were also analyzed for pod set % (PSP), and yield-related traits [number of total pods plant^–1^ (NPP), seed yield plant^–1^ (SYP), single seed weight plant^–1^ (SSWP), biological mass]. The screening experiment (outdoors; conducted for two consecutive years) comprised of 10 pots for each of 39 genotypes (three plants pot^–1^) grown in triplicate in a randomized block design. The validation experiment CRBD comprised of five pots for each selected genotypes (10 heat-tolerant and 10 heat-sensitive; three plants pot^–1^) grown in triplicate in a complete randomized block design.

### Physiological Traits

#### Electrolyte Leakage %

The leaves were assessed for membrane damage (as EL) to measure the permeability of cell membranes. Fresh leaves (100 mg) from the topmost branches were collected and washed with deionized water to remove surface-adhered electrolytes. The leaf tissue was placed in closed glass vials containing 10 mL deionized water and incubated at 25°C on a rotary shaker for 24 h. Electrical conductivity of the solution (*L*_1_) was measured with a conductivity meter (ELICO CM 180, Hyderabad, India), expressed in mmhos g^–1^ dry weight (DW). The samples were then heated in a water bath at 120°C for 20 min before measuring final electrical conductivity (*L*_2_) after equilibration at 25°C (Lutts et al., 1996). Electrolyte leakage was defined as EL (%) = (*L*_1_/*L*_2_) × 100.

#### Stomatal Conductance

The stomatal conductance of fully expanded leaves (from the second or third branches from the top) was measured using a portable leaf porometer (model SC1, Decagon Devices, Pullman, WA, United States) at 11:00 h at the end of the stress period and expressed as mmol m^–2^ s^–1^ (Awasthi et al., 2014).

#### Chlorophyll Content (SPAD)

The chlorophyll (CHL; as SPAD value) of a tagged leaf was measured using a SPAD chlorophyll meter between 10.00 and 11.00 h using an Apogee-SPAD meter on alternative days for 2 weeks from 30 DAS (days after sowing).

#### Malondialdehyde

Lipid peroxidation of membranes was estimated in terms of MDA concentration, a lipid peroxidation product, following the method of Heath and Packer (1968). Fresh plant tissue (500 mg) was homogenized in 0.1% trichloroacetic acid (TCA), followed by centrifugation at 15,000 rpm for 5 min. A fraction of the supernatant (0.1 mL) was reacted with 0.5% thiobarbituric acid (4 mL) prepared in 20% TCA. The mixture was heated at 95°C for 30 min and then quickly cooled in an ice bath, followed by centrifugation at 10,000 rpm for 10 min at 4°C. The supernatant was used to measure absorbance at 532 nm. The MDA content was calculated using its extinction coefficient (155 mM^–1^ cm^–1^) and expressed as nmol g^–1^ DW.

#### Leaf Photosynthetic Function

Photochemical efficiency was measured as chlorophyll fluorescence using the dark-adapted test of the modulated chlorophyll fluorometer OS1-FL (Opti-Sciences, Tyngsboro, MA, United States) at 11:00 h (Awasthi et al., 2014).

### Reproductive Function

For assessing reproductive function, flowers were collected 15 days after exposure to stress.

#### Pollen Germination

*In vitro* pollen germination experiments were conducted using pollen grains collected from five flowers genotype^–1^ in three replications, as described by Brewbaker and Kwack (1963). The germination medium comprised 10% sucrose, 990 mM potassium nitrate (pH 6.5), 1.3 mM calcium nitrate, 1.64 mM boric acid, and 812 mM magnesium sulfate. Pollen grains are considered germinated when the diameter of the tube exceeds the diameter of the pollen grain. PGP was calculated from at least 100 pollen grains per replicate (Kaushal et al., 2013).

#### Pollen Viability

For PVP, pollen grains were collected from flowers that opened on the same day and pooled (Alexander, 1969), before adding 0.5% acetocarmine/Alexander stain. Viable pollen grains were selected based on shape and size (spherical or triangular) and the intensity of stain uptake (Kaushal et al., 2013). The observations were recorded for at least ten microscopic fields.

### Yield Traits

Pod set %, NPP, SSWP, and SYP significantly varied (*P* < 0.01) among genotypes (Table 1). Under HS, these traits had high heritability, with 90.7, 89.4, and 89.7% in 2017–2018 and 90, 90.4, and 90.1% in 2018–2019, respectively, (Table 2).

**TABLE 1:** ANOVA for various traits recorded in chickpea genotypes grown outdoors in 2017–2018 and 2018–2019, and in a growth chamber under heat stress.

	Outdoor environment 2017–2018			Outdoor environment 2018–2019			Growth chamber		
	*T*	*G*	*T* × *G*	*T*	*G*	*T* × *G*	*T*	*G*	*T* × *G*
Electrolyte leakage	<0.01	<0.01	<0.01	<0.01	<0.01	<0.01	<0.01	<0.01	<0.01
Chlorophyll	<0.01	<0.01	<0.01	<0.01	<0.01	<0.01	<0.01	<0.01	<0.01
Photosynthetic efficiency (Fv/Fm)	<0.01	<0.01	ns	<0.01	<0.01	<0.01	<0.01	<0.01	<0.01
Stomatal conductance	ns	<0.01	<0.01	<0.01	<0.01	<0.01	<0.01	<0.01	<0.01
Malondialdehyde	<0.01	<0.01	<0.01	<0.01	<0.01	<0.01	<0.01	<0.01	<0.01
Pollen viability (%)	<0.01	<0.01	<0.01	<0.01	<0.01	<0.01	<0.01	<0.01	<0.01
Pollen germination (%)	<0.01	<0.01	<0.01	<0.01	<0.01	<0.01	<0.01	<0.01	<0.01
Pod set (%)	<0.01	<0.01	<0.01	<0.01	<0.01	<0.01	<0.01	<0.01	<0.01
Number of pods/plants	<0.01	<0.01	<0.01	<0.01	<0.01	<0.01	<0.01	<0.01	<0.01
Single seed weight per plant	<0.01	<0.01	<0.01	<0.01	<0.01	<0.01	<0.01	<0.01	<0.01
Seedyield per plant	<0.01	<0.01	<0.01	<0.01	<0.01	<0.01	<0.01	<0.01	<0.01
Biological mass	<0.01	<0.01	<0.01	<0.01	<0.01	<0.05	<0.01	<0.01	<0.01

*ns, non significant.*

**TABLE 2:** General statistics of various traits in chickpea genotypes under heat stress environment.

Traits	Mean	SE	Range	Heritability *h*^2^ (%)
**Heat-stress environment, 2017–2018**				
Electrolyte leakage (%)	22.4	1.3	15.33–27.33	91.5
SPAD chlorophyll	14.47	0.6	12.3–18.13	92.1
Photosynthetic efficiency (Fv/Fm)	0.45	0	0.31–0.62	90
Stomatal conductance	427.4	19.3	308.7–571.9	94.8
Malondialdehyde	43.5	3.2	22.8–57.4	94.3
Pollen viability (%)	37.77	3.5	24–58.7	92.4
Pollengermination (%)	44.6	3.3	24.5–66.17	94.8
Pod set (%)	30.2	3	20.77–45.63	90.7
Number of podsplant^–1^	12.58	1.3	5.33–20.67	89.4
Single seed weight (g)	0.19	0	0.11–0.29	93.2
Seed yield plant^–1^ (g)	2.47	0.4	0.71–4.94	89.7
Biological mass (g)	4.51	0.4	2.15–7.4	87.7
**Heat-stress environment, 2018–2019**				
Electrolyte leakage (%)	22.77	1.7	14.33–27.53	89.3
Chlorophyll	14.9	0.7	12.77–19	91.5
Photosynthetic efficiency (Fv/Fm)	0.46	0	0.33–0.63	90.6
Stomatal conductance	410.5	15.3	307.8–518.03	95.5
Malondialdehyde	44.17	3.2	22.77–58.03	95.5
Pollen viability (%)	36.74	3.1	23.47–55.83	94.5
Pollengermination (%)	42.85	3.9	21.37–64	93.3
Pod set (%)	31.36	3	22.83–45.73	90
Number of pods plant^–1^	12.38	1.1	6.67–18.67	90.4
Single seed weight (g)	0.18	0	0.11–0.24	95
Seedyield plant^–1^ (g)	2.36	0.3	0.66–4.73	90.1
Biological mass (g)	4.45	0.3	2.62–5.61	84.8
**Heat-stress environment under growth chamber**				
Electrolyte leakage (%)	40.3	2.4	23.8–55.43	96.6
Chlorophyll	10.8	0.8	6.13–14.83	94.8
Photosynthetic efficiency (Fv/Fm)	0.51	0	0.34–0.64	96.8
Stomatal conductance	340.4	22.1	273.6–430.8	88
Malondialdehyde	21.7	1.3	14.8–32.4	96.8
Pollen viability (%)	0.51	0	0.34–0.64	96.8
Pollengermination (%)	45.6	2.2	32.07–61.07	96.2
Pod set (%)	51.38	1.7	33.63–64.6	98.4
Number of pods plant^–1^	7.8	0.7	4.67–11.33	91.4
Single seed weight (g)	0.27	0.1	0.1–0.93	95.7
Seed yield plant^–1^ (g)	1.86	0.2	0.68–3.81	94.6
Biological mass (g)	4.1	0.2	3.17–4.82	90.8

### Statistical Analyses

The plants were raised in outdoor and growth chamber environments using CRBD. Data were analyzed as a two-factorial (temperature and genotypes) experimental design using AGRISTAT statistical software (ICAR Research Complex, Goa, India). Standard errors and least significant differences (*P* < 0.05) for genotypes, treatments, and their interaction were computed. Phenotypic correlations were estimated to determine trait associations in GenStat 15. Using the R package cluster (Patterson and Thompson, 1971), the Euclidean dissimilarity matrix was constructed using all the traits and the accessions were clustered following Ward’s method. Similarly, using the R package factoextra, the principal component analysis was performed. Heat map analysis was done as per Babicki et al. (2016).

## Results

Significant genetic variability for the tested traits was observed under normal and HS environment in both years (2017–2018 and 2018–2019). In accordance with the various tested physiological parameters and yield-related traits in the screening experiments, genotypes GNG2171, GNG1969, GNG1488, PantG186, CSJ515, RSG888, RSG945, RVG202, and GNG469 were identified as promising under HS in both years. In contrast, genotypes ICC10685, ICC96030, DCP92-3, PDG3, PDG4, GL15026, and GL15017 were identified as highly heat-sensitive due to their low pod set, SYP and other physiological parameters in both years.

Accordingly, ten heat-tolerant and ten heat-sensitive lines were selected from the set of 39 genotypes and further validated in a growth chamber under normal and heat-stressed conditions. Among the ten heat-tolerant lines, GNG1969, GNG1488, PantG186, RSG888, CSJ315, and GNG1499 exhibited high heat tolerance evidenced by small reductions in PVP, PGP, PSP, and SYP. Among the ten heat-sensitive lines, CSG8962, DCP92-3, IPC13-8, IPC14-9, and RSG931 exhibited high heat sensitivity evidenced by large reductions in these traits.

### Physiological Traits

#### Electrolyte Leakage %

Heat stress significantly (*P* < 0.01) affected EL, a measure of membrane damage (Supplementary Figure 2 and Table 1). Under HS, GNG 1969 (15.3%), GNG 1499 (16.4%), and Pant G186 (15.6%) had the lowest EL values in 2017–2018, and GNG 469, GNG 217, and Pant G186 had the lowest EL values in 2018–2019, similar to the heat-tolerant check ICC92944 (Supplementary Figure 2). EL had medium–high heritability under normal conditions (65.5 and 56.5%) and high heritability under HS (91.5 and 89.3%; Table 2). The heritability (h^2^; %) of other traits is listed in Table 2.

#### Stomatal Conductance

Stomatal conductance (Supplementary Figure 3) varied among genotypes (*P* < 0.01; Table 1). Under normal conditions, GL13042 had the maximum value (485.7 mmol m^–2^ s^–1^) in 2017–2018, and Pant G186 had the maximum value (511.70 mmol m^–2^ s^–1^) in 2018–2019. Under HS, GNG1969 had the highest stomatal conductance (565.6) in 2017–2018, and GNG1488 (513.17) and GNG469 (511.73) had the highest in 2018–2019.

#### Chlorophyll (SPAD)

SPAD chlorophyll (Supplementary Figure 4) content declined drastically under HS and varied among genotypes (*P* < 0.01; Table 1). Mean SPAD values were 23.28 and 23.77 under normal conditions and 14.47 and 14.9 under HS in 2017–2018 and 2018–2019, respectively. GNG2171 (18.13) and GNG2299 (19) had the highest chlorophyll contents under HS in 2017–2018 and 2018–2019, respectively.

#### Chlorophyll Fluorescence

Chlorophyll fluorescence (Fv/Fm; Supplementary Figure 5) declined significantly under HS and varied among genotypes (*P* < 0.01; Table 1). Mean Fv/Fm values were 0.74 and 0.73 under normal conditions and 0.45 and 0.46 under HS in 2017–2018 and 2018–2019, respectively. GNG469 (0.62) and GNG1488 (0.63) had the highest Fv/Fm values under HS in 2017–2018 and 2018–2019, respectively.

#### Malondialdehyde

Malondialdehyde content varied significantly (*P* < 0.01) among genotypes (Supplementary Figure 6 and Table 1), with mean values of 14.06 and 14.25 nmol g^–1^ DW under normal conditions and 43.5 and 44.17 under HS in 2017–2018 and 2018–2019, respectively. Under HS, GNG1488, GNG 1581, GNG 2207, RSG 888, and RSG 931 had the lowest Malondialdehyde contents (22.80–25.43) in 2017–2018, while GNG2207, GNG1488, GNG1499, and GNG663 had the lowest (22.8–23.77) in 2018–2019.

### Reproductive Traits

#### Pollen Viability (%)

A considerable amount of genetic variability (*P* < 0.01; Table 1) was recorded for PVP (Supplementary Figure 7) and PGP (Supplementary Figure 8). Mean PVP values were 75.3 and 73.7 under normal conditions and 37.77 and 36.74 under HS. Maximum PVP values under normal conditions were recorded in GNG1499 and ICCV10 in 2017–2018 and 2018–2019, respectively. Under HS, PVP in GNG 2171 (24.45%) and RSG 931 (26.03%) in 2017–2018 and GNG 2144 (23.7%), GNG 469 (24%), and PantG 186 (23.9%) in 2018–2019 declined less than the heat-tolerant check ICCV 92944 (37.2 and 59.7%, respectively), relative to normal conditions.

#### Pollen Germination (%)

Under HS, PGP in GNG2171 (12.30%), RSG888 (12.16%), and GNG469 (13.96%) in 2017–2018 and GNG1581 (12.34%), CSJ515 (13.44%), and GNG2171 (13.57%) in 2018–2019 declined less than the heat-tolerant check ICCV92944 (43.93 and 53.83%, respectively), relative to normal conditions (Figure 8).

### Yield Traits

**FIGURE 1:**
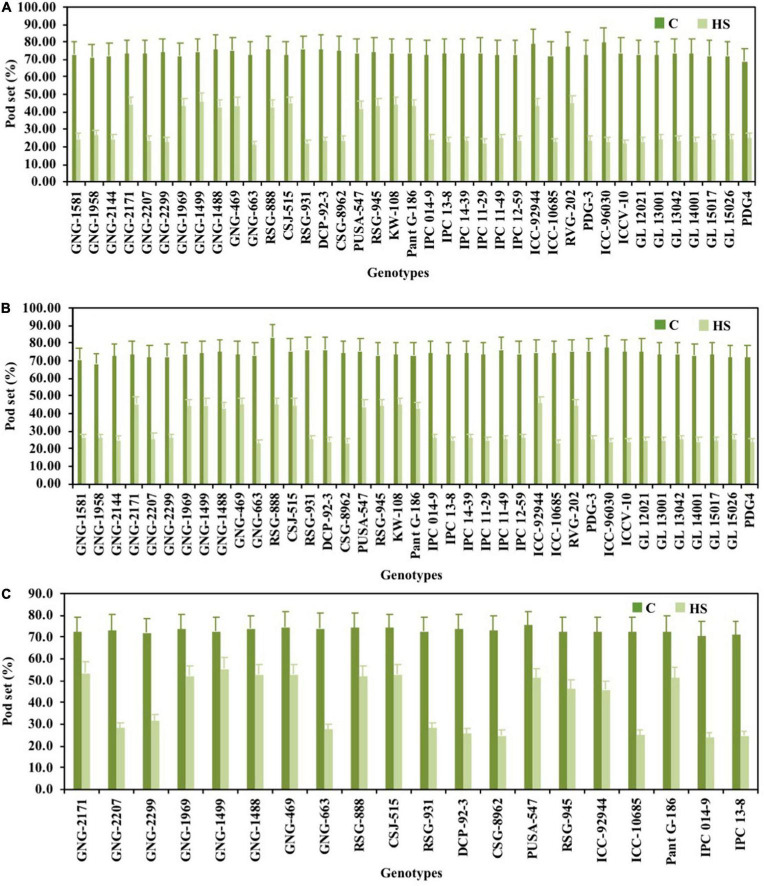
Pod set (%) of chickpea genotypes under control (normal-sown; C) and heat stress (late-sown; HS) environment during 2017–2018 **(A)**, 2018–2019 **(B)** and in control environment of growth chamber (GC; C-control; HS-heat stress; **C)**. LSD values (*P* < 0.05); genotype × treatment: 9.3 (2017–2018), 11.3 (2018–2019), 11.7 (GC). Values are means + SE. (*n* = 3).

**FIGURE 2:**
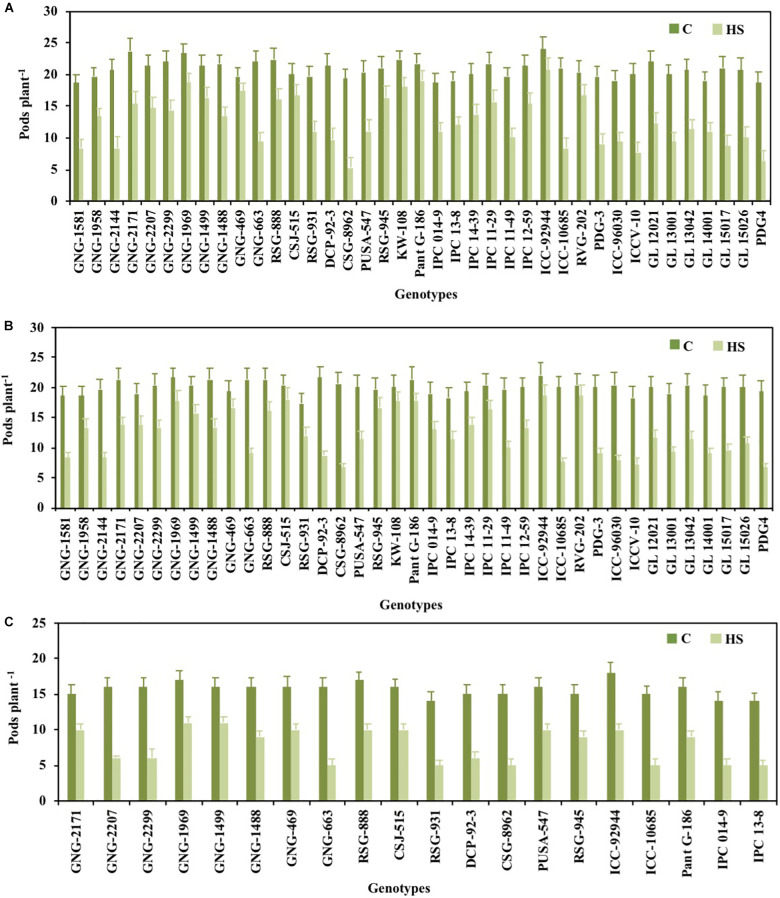
Pod number plant^– 1^ of chickpea genotypes under control (normal-sown; C) and heat stress (late-sown; HS) environment during 2017–2018 **(A)**, 2018–2019 **(B)** and in control environment of growth chamber (GC; C-control; HS-heat stress; **C)**. LSD values (*P* < 0.05); genotype × treatment: 2.9 (2017–2018), 3.6 (2018–2019), 3.1 (GC). Values are means + SE. (*n* = 3).

**FIGURE 3:**
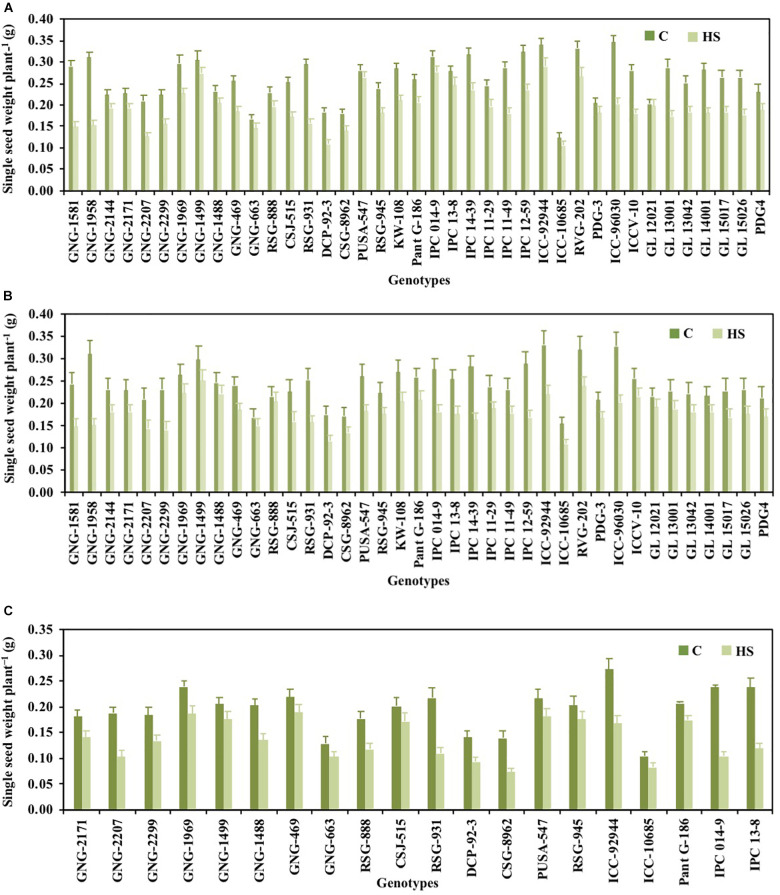
Single seed weight of chickpea genotypes under control (normal-sown; C) and heat stress (late-sown; HS) environment during 2017–2018 **(A)**, 2018–2019 **(B)** and in control environment of growth chamber (GC; C-control; HS-heat stress; **C)**. LSD values (*P* < 0.05); genotype × treatment: 0.15 (2017–2018), 0.17 (2018–2019), 0.14 (GC). Values are means + SE. (*n* = 3).

**FIGURE 4:**
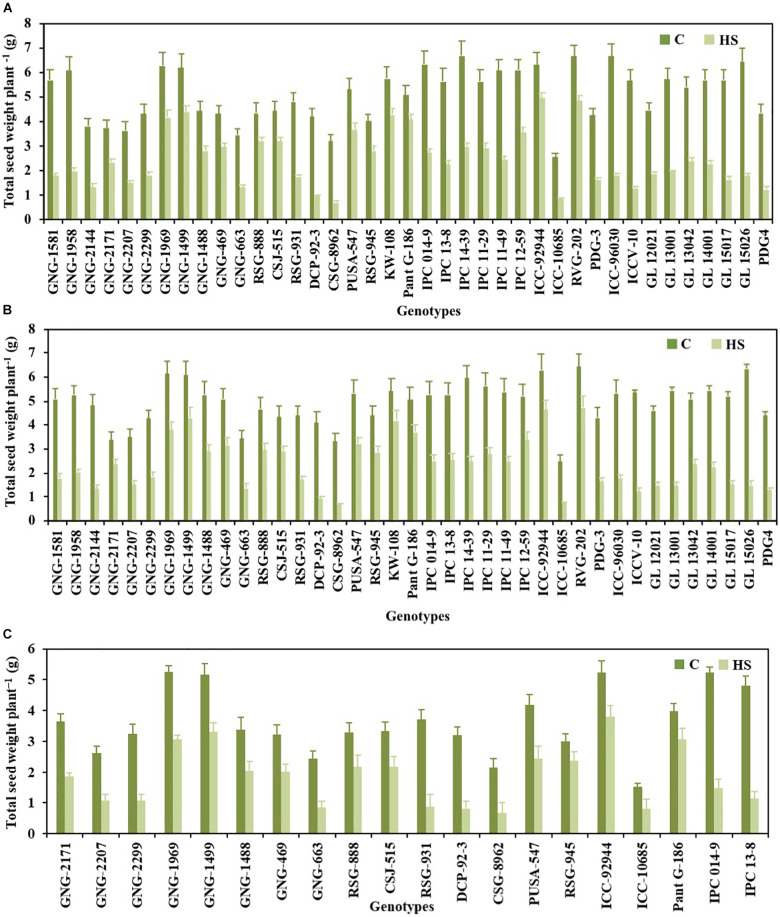
Seed weight plant^– 1^ of chickpea genotypes under control (normal-sown; C) and heat stress (late-sown; HS) environment during 2017–2018 **(A)**, 2018–2019 **(B)** and in control environment of growth chamber (GC; C-control; HS-heat stress; **C)**. Values are means + SE. (*n* = 3).

Pod set % (Figure 1), NPP (Figure 2), SSWP (Figure 3), and SYP (Figure 4) significantly varied (*P* < 0.01) among genotypes (Table 1). Under HS, these traits had high heritability, with 90.7, 89.4, and 89.7% in 2017–2018 and 90, 90.4, and 90.1% in 2018–2019, respectively, (see Table 2 for heritability of other traits).

GNG2144 (45.63%), GNG (44.97%), GNG1958 (44.80%), KWR (44.4%), RSG888 (43.63%), and GNG1488 (42.6%) in 2017–2018 and GNG1499 (43.97%), GNG1488 (44.875), GNG469 (44.97%) genotypes, and PantG186 (45.17%) in 2018–2019 had the highest PSP under HS, relative to heat-tolerant check ICCV92944 (24.6%; Figure 1).

GNG1581 (20.67), GNG2299 (19), RSG888 (17.3), and GNG469 (16.67) in 2017–2018 and GNG1581 (18.67), GNG2171 (16.67), and GNG663 (18) in 2018–2019 had the highest NPP under HS (Figure 2).

GNG1581 (0.29), GNG2144 (0.27), and GNG1958 (0.27) in 2017–2018 and GNG1581 (0.24) and GNG2144 (0.25) in 2018–2019 had higher single seed weights plant^–1^ than the heat-tolerant check ICC92944 (0.15 and 0.17 *g*, respectively) under HS (Figure 3).

Pant G186 (19.7%) in 2017–2018 and KWR108 (22.60%) in 2018–2019 had somewhat similar reductions in SYP as the heat-tolerant check ICCV92944 (21.6 and 26.15%, respectively) under HS, relative to normal conditions (Figure 4).

**IMAGE 1:**
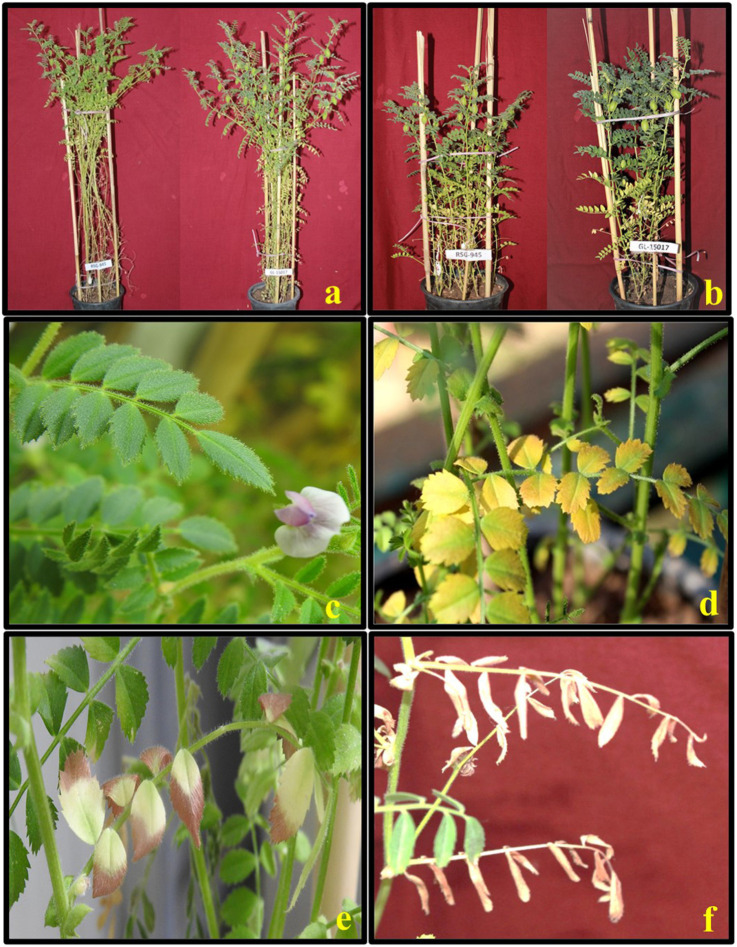
Morphological symptoms of heat stress (HS) observed on in chickpea plants, showing plant height; under in the control condition **(a)**, reduced plant height; under the HS environment **(b)**, healthy leaves in the; under control condition **(c)**, leaf chlorosis under HS **(d)**, and leaf scorching ofleaves **(e)**, and leaf bleaching **(f)** ofleaflets due to photooxidation under HS **(e,f)**.

**IMAGE 2:**
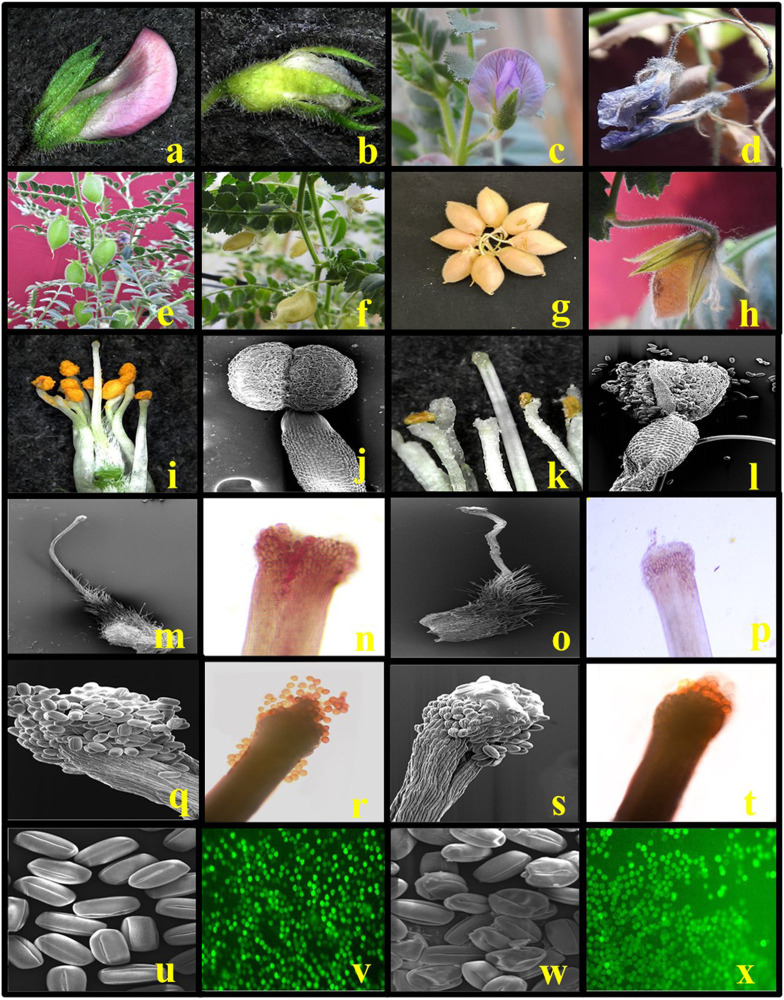
Effects of heat stress (HS) in chickpea (reproductive phase) in comparison to control plants. Healthy flower bud under in the control condition **(a)**, aborted bud under HS **(b)**, healthy flower under in the control condition **(c)**, aborted flower under HS **(d)**, healthy pod under in the control condition **(e)**, aborted pod under HS **(f)**, mature filled pod under in the control condition **(g)**, unfilled mature pod under HS **(h)**, dissected flower showing the healthy anther under in the control condition **(i,j)**, distorted exposed anther under HS **(k,l)**, healthy style and stigma under in the control condition **(m,n)**, noticeable distorted shriveled stigma and style under HS **(o,p)**, healthy flower pollen load under in the control condition **(q,r)**, reduced pollen load under HS **(s,t)**, healthy viable pollen grains under in the control condition **(u,v)**, and distorted and shriveled pollen grains **(w,x)** as a sign of HS sensitivity to heat stress (HS).

Images 1, 2 illustrate the effects of HS on vegetative and reproductive components of chickpea plants.

### Validation of Selected Genotypes Under Growth Chamber

The validation experiment in the growth chamber revealed significant differences (*P* < 0.01) between the ten heat-tolerant and ten heat-sensitive genotypes for the assessed traits (Table 1). On the basis of chlorophyll content and stomatal conductance, genotypes GNG1488 (chlorophyll: 17.83 mg g^–1^ DW; Supplementary Figure 4C) and RSG888 (stomatal conductance; 388.2 mmol m^–2^ s^–1^; Supplementary Figure 3C were identified as heat-tolerant. Likewise, GNG469 had high PVP (58.9%; Supplementary Figure 7C) and chlorophyll fluorescence values (Supplementary Figure 5C) and RSG888 had high PGP (64.6%; Supplementary Figure 8C) under HS. Genotype GNG1499 had the highest NPP (11.3; Figure 2C) under HS. The SYP of GNG1499 (16.9%), GNG 469 (22.6%), Pant G186 (6.8%), and RSG888 (27.5%) declined the least under HS (Figure 4C). Thus, the controlled environment study validated the heat tolerance of the selected genotypes based on various leaf-based reproductive and yield traits.

**FIGURE 5:**
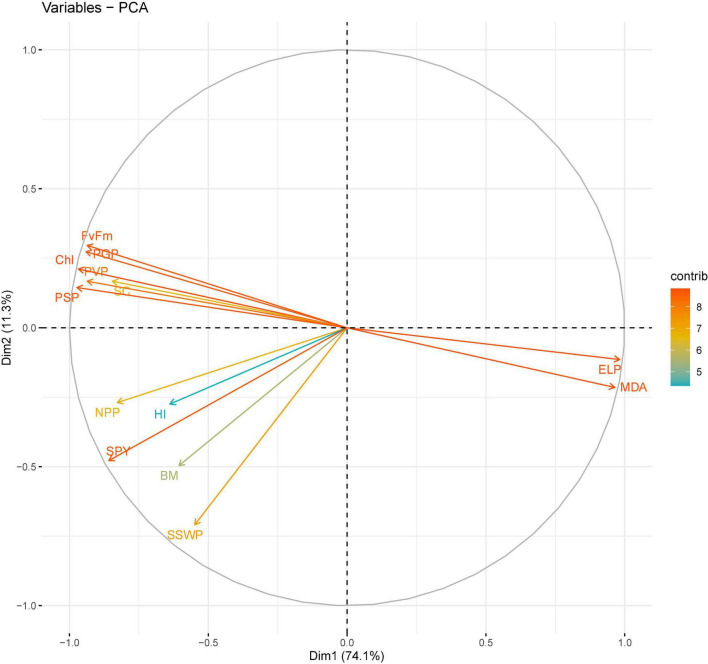
Principal component analysis (PCA) of various traits in chickpea genotypes under heat stress in 2017–2018.

**FIGURE 6:**
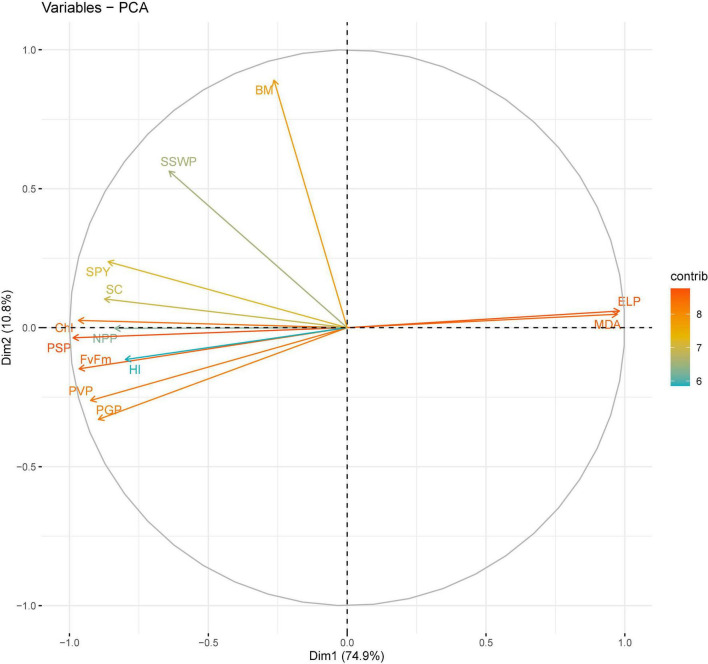
Principal component analysis (PCA) of various traits in chickpea genotypes under heat stress in 2018–2019.

**FIGURE 7:**
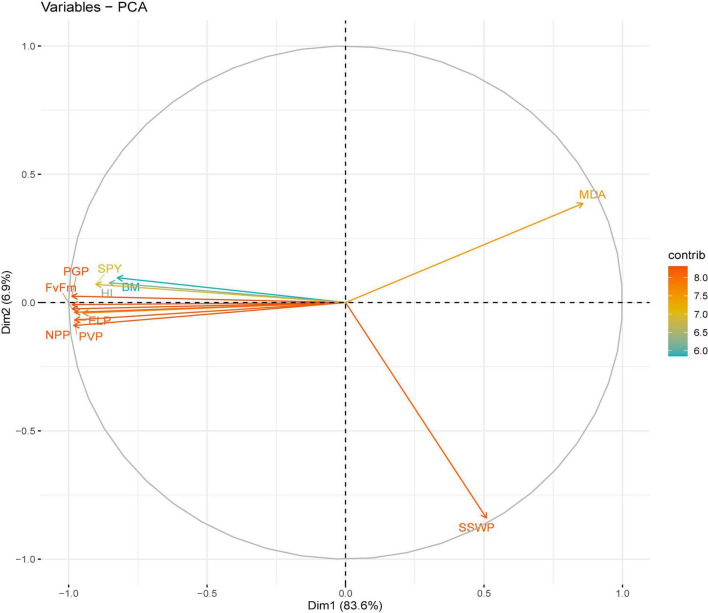
Principal component analysis (PCA) of various traits in chickpea genotypes under heat stress in a growth chamber.

**FIGURE 8:**
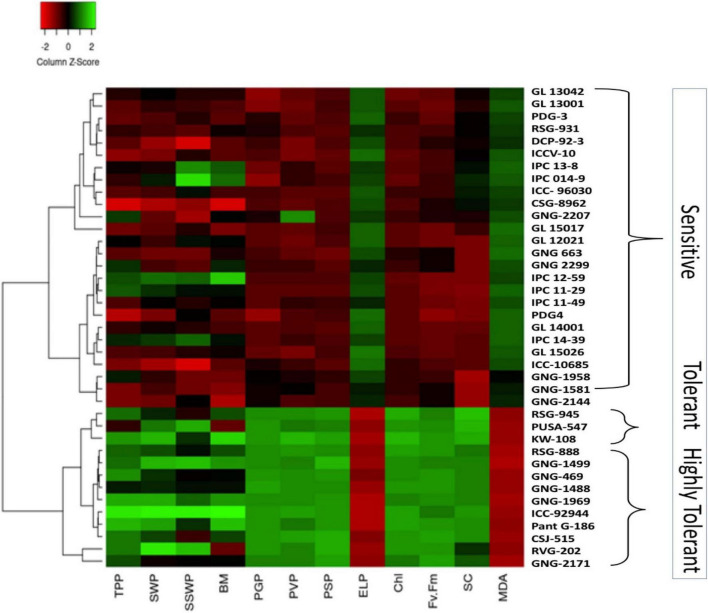
Heat map based on the response of chickpea genotypes to heat stress in 2017–2018.

### Correlation Analysis

Electrolyte leakage significantly correlated with NPP and SYP in all growth environments (Table 3). Similarly, significant negative correlations occurred between malondialdehyde and yield traits. Other leaf-based traits such as chlorophyll, photosynthetic efficiency, and stomatal conductance had strong positive correlations with NPP and SYP. Pollen-based traits such as PVP and PGP had significant positive correlations with yield traits. PSP strongly correlated with NPP and SYP (Table 3). In general, all of the measured traits, except malondialdehyde and EL, had strong positive correlations with NPP and SYP.

**TABLE 3:** Correlation coefficients of various traits with yield traits in plants under heat stress environment.

	Outdoor environment 2017–2018		Outdoor environment 2018–2019		Growth chamber	
	Number of pods plant^–1^	Seedyield plant^–1^	Number of pods plant^–1^	Seedyield plant^–1^	Number of pods plant^–1^	Seedyield plant^–1^
Electrolyte leakage (%)	–0.77**	–0.79**	–0.77**	–0.77**	–0.79**	–0.77**
Chlorophyll	0.73**	0.73**	0.76**	0.77**	0.96**	0.86**
Photosynthetic efficiency (Fv/Fm)	0.68**	0.65**	0.74**	0.73*	0.97**	0.85**
Stomatal conductance	0.58**	0.61**	0.62**	0.67**	0.92**	0.83**
Malondialdehyde	–0.72**	–0.73**	–0.74**	0.75**	–0.85**	–0.73**
Pollen viability (%)	0.77**	0.72**	0.73**	0.67**	0.98**	0.84**
Pollen germination (%)	0.71**	0.68**	0.72**	0.67**	0.97**	0.84**
Pod set (%)	0.74**	0.77**	0.78**	0.80**	0.98**	0.81**
Number of pods plant^–1^	1	0.84**	1	0.85**	1	0.87**
Single seed weight	0.46**	0.77**	0.50**	0.72**	–0.42**	–0.47**
Biological mass	0.80**	0.72**	0.70**	0.37*	0.80**	0.73**
Harvest index	0.56**	0.75**	0.82**	0.91**	0.81**	0.98**

*Significance at 5% and ** Significance at 1%.

### Principal Component Analysis

In 2017–2018, four principal components, correlated to 12 traits, accounting for 96.6% of the total variability under HS. The individual contribution of each component was 74, 11.3, 7.7, and 3.4% (Figure 5). For PC1, major contributors are EL (0.3163) and malondialdehyde (0.31062) and chlorophyll (-0.31184) contributed the most negatively to PC1. For PC2, chlorophyll fluorescence (0.244) and PGP (0.225) had the greatest positive contributions, and SSWP (-0.584) and biomass (-0.409) had the greatest negative contributions. For PC4, SC (0.628) and SSWP (0.481) had the greatest positive contribution, and NPP (-0.488) had the greatest negative contribution.

In 2018–2019, four principal components, correlated to 12 traits, accounted for 96% of the total variability under HS. The individual contribution of each component was 74.5, 10.7, 7.2, and 3% (Figure 6). Analysis of the factor loadings of the characters in the retained PCs revealed that EL (0.314) and malondialdehyde (0.312) contributed most positively, and PSP (-0.316) contributed most negatively to PC1. For PC2, biomass (0.75) and SSWP (0.476) had the greatest positive contributions and PGP (-0.27) had the greatest negative contribution. For PC3, NPP (0.347), SYP (0.416), and harvest index (0.58) had the greatest positive contributions, and stomatal conductance (-0.278) had the greatest negative contribution. For PC4, NPP (0.53) and biomass (0.38) had the greatest positive contribution and SSWP (-0.68) had the greatest negative contribution.

For the growth chamber environment, the extracted sums of squares loadings and component correlation matrix revealed three principal components correlated to all evaluated traits accounting for 94.7% of the total variability. The individual contribution of each component was 83.6, 6.9, and 4.2%. Analysis of the factor loadings of the characters in the retained PCs revealed that malondialdehyde (0.25) had the highest positive value and PGP (-0.2997) had the highest negative value for PC1 (Figure 7). For PC2, malondialdehyde (0.408) and biomass (0.102) had the highest positive values and SSWP (-0.886) had the highest negative value. For PC3, harvest index (0.68) and SYP (0.497) had the highest positive values, whereas biomass (-0.409) had the highest negative value.

### Cluster Analysis for Identifying Heat-Tolerant Chickpea Genotypes Based on Physiological Yield Attributes in an Outdoor Environment

In 2017–2018, the heat map based on physiological attributes and yield responses of 39 chickpea genotypes to HS in an out-door environment revealed three clusters. Cluster I contained highly heat-sensitive genotypes based on high SYP reduction, PDG4 (71.53%), GL15026 (72.23%), GL15017 (71.28%), DCP92-3 (76.75%), CSG8962 (77.73%), and ICC10685 (66.77%; Figure 8). Cluster II contained heat-tolerant genotypes RSG945 (29.64%), KWR108 (25.1%), and PUSA547 (30.57%). Cluster III contained the most heat-tolerant genotypes CSJ515 (28.1%), RSG888 (25.4%), PantG186 (19.7%), ICCV92944 (21.6%), and GNG1499 (29.5%), based on lower seed yield per plant reduction.

**FIGURE 9:**
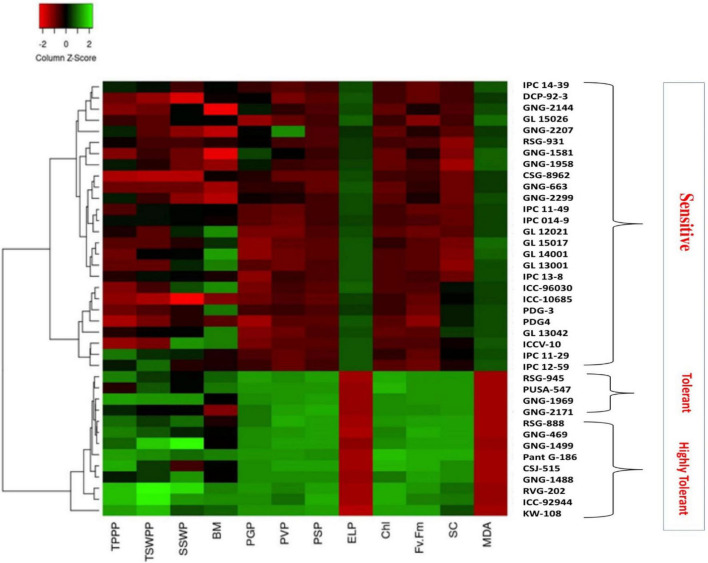
Heat map based on the response of chickpea genotypes to heat stress in 2018–2019.

Similarly, in 2018–2019, the heat map comprised three clusters (Figure 9). Cluster I contained highly heat-sensitive genotypes GL15026 (76.6%), GL13001 (73.3%), ICC10685 (70.8%), DCP92-3 (77.9%), and CSG8962 (80%). Cluster II contained heat-tolerant genotypes RSG945 (34.7%), GNG1969 (38.1%), and PUSA547 (39.4%). Cluster III contained highly heat-tolerant genotypes CSJ515 (32.9%), RSG888 (36.9%), PantG186 (26.7%), ICCV92944 (26.2%), and GNG1499 (29.3%).

### Identifying Heat-Tolerant Genotypes in a Controlled Environment

**FIGURE 10:**
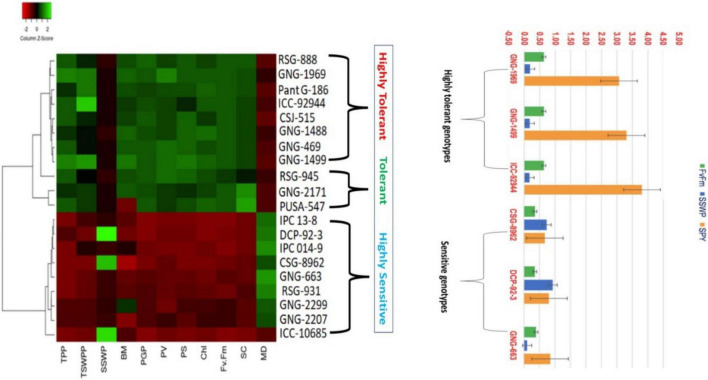
Heat map based on the response of chickpea genotypes to heat stress in a growth chamber.

The heat map based on the phenotypic responses of 20 selected genotypes to HS in a growth chamber revealed three clusters based on seed yield reduction (single plant) under normal and HS conditions (Figure 10). Based on low reduction of seed yield per plant, five of the ten selected heat-tolerant lines—RSG945 (20.80%), PantG186 (23.2%), ICCV92944 (27.17%), RSG888 (33.67%), and CSJ315 (34.57%)—exhibited high heat tolerance and five of the ten selected heat-sensitive lines—RSG931 (76.4%), IPC13-8 (76.1%), DCP92-3 (74.58%), IPC14-9 (71.7%), and CSG8962 (68.58%)—exhibited high heat sensitivity.

## Discussion

Heat stress during the reproductive stage, especially pollen development and fertilization, pod set, and grain filling, significantly reduces yield in various crops, including chickpea (Awasthi et al., 2014), wheat (Yang et al., 2002), lentil (Sita et al., 2017; Bhandari et al., 2020), wheat (Bheemanahalli et al., 2019), and pea (Jiang et al., 2020; Mohapatra et al., 2020). Therefore, capturing genetic diversity for various traits of physiological and breeding importance is a prime objective for developing heat-tolerant chickpea genotypes.

Here, we phenotyped 39 chickpea genotypes to identify sources of HS tolerance by exposing them to HS in the field and a growth chamber during heat-sensitive stages. We identified potential heat-tolerant chickpea donors with high pod set and grain yield, high chlorophyll content, PVP, PGP, photosystem II efficiency, and low MDA and EL% under HS.

High CHL content, photosystem II function, and stomatal conductance are important traits for selecting photosynthetically efficient genotypes under HS (Chaudhary et al., 2020). The heat-tolerant chickpea genotypes in the present study showed more chlorophyll content than heat-sensitive genotypes under high temperature environment of outdoor and growth chamber; these findings are similar to those reported previously in lentil plants subjected to HS (Sita et al., 2017; Bhandari et al., 2020). Previously, an average of 8% reduction in chlorophyll variable fluorescence, an important trait measuring injury to photosynthesis, was noted under HS condition, compared to normal environment, in pea (McDonald and Paulsen, 1997). Similarly, based on chlorophyll fluorescence induction traits, Petkova et al. (2007) screened 12 common bean lines for heat tolerance; “Ranit” and “Nerine” RS lines were identified to be heat tolerant on the basis of chlorophyll fluorescence. Improved functioning of stomatal conductance (gs), lower EL % and high retention of relative water content allowed in identifying 10 lentil genotypes viz., IG2507, IG3263, IG3297, and IG3312 under HS environment (Sita et al., 2017). Thus, in our study, we identified sufficient genetic variability for these traits under normal and HS conditions in the field and growth chamber for selecting heat-tolerant chickpea genotypes.

Heat stress decreases PGP and PVP in rice (Coast et al., 2016), wheat (Bheemanahalli et al., 2019), peanut (Kakani et al., 2002), sorghum (Sunoj et al., 2017), common bean (Soltani et al., 2019; Vargas et al., 2021), and chickpea (Bhandari et al., 2020). High values of these traits indicate better reproductive function and thus high grain yield. The wide range of PGP (26.2–66.1% in 2017–2018 and 21.3–64% in 2018–2019) and PVP (24–58.7% in 2017–2018 and 23.4–55.8% in 2018–2019) under HS suggests great scope for developing heat-tolerant chickpea genotypes. Based on these two traits, GNG469 and CSJ515 genotypes exhibited higher heat tolerance than the heat-tolerant check ICCV 92944, and could be used as donor parents to improve heat tolerance in chickpea. Several researchers have used *in vitro* pollen germination to screen for heat tolerance in various crops such as peanut (Kakani et al., 2002) and cotton (Kakani et al., 2005; Song et al., 2015), mung bean (Sharma et al., 2016) and common bean (Vargas et al., 2021).

Serious losses in yield and yield-related traits (biomass, pod set, seed weight, and SYP) have been reported in various crops under HS such as winter wheat (Wheeler et al., 1996; Prasad and Djanaguiraman, 2014; Bheemanahalli et al., 2019), cowpea (Ehlers and Hall, 1998), common bean (Soltani et al., 2019), pea (Tafesse et al., 2019; Jiang et al., 2020), and lentil (Bhandari et al., 2020). HS during the reproductive stage, especially pollen development and fertilization, damaged pod and seed setting processes and thus reduced grain yield in many crops (see review by Prasad et al. (2017), for instance in common bean (Rainey and Griffiths, 2005; Vargas et al., 2021), and pea (Jiang et al., 2020). The wide range of PSP (21.7–44.9% in 2017–2018 and 22.8–45.7% in 2018–2019) under HS in the present study indicates great scope for developing chickpea genotypes involving under HS. In addition, PSP positively correlated with PVP and PGP under HS, indicating that improving these traits could lead to higher pod set% and thus improve grain yield under HS environment. Singh et al. (2015) also reported positive associations between PSP with PGP under HS in sorghum. Likewise, negative impact of HS on pollen fertility and seed setting has been reported in common bean (Soltani et al., 2019) and in lentil (Sita et al., 2017). Significant reductions in grain yield after HS exposure during grain filling have been reported in various crops such as wheat (Yang et al., 2002; Aziz et al., 2018), common bean (Rainey and Griffiths, 2005) including chickpea (Bhandari et al., 2020). Seed weights declined by up to 59.7% in 2017–2018 and 54.8% in 2018–2019 under HS in the field. Similar reductions (up to 50%) have been reported in wheat (Yang et al., 2002; Liu et al., 2016; Barber et al., 2017) and 37% (during the year 2016) and 26% (during the year 2017) in common bean (Vargas et al., 2021) and 16% in pea (Tafesse et al., 2019) under field condition under HS. Previously, Rainey and Griffiths (2005) also advocated reduction in seed number (83%), pod number (63%), mean seed weight (47%), and seeds pod^–1^ (73%) in common bean subjected to 33°C/30°C under greenhouse condition. In case of pea, based on high pod number, seed retention and improved seed yield under HS, genotypes “40–10,” “Naparnyk,” and “CDC Meadow” were found to be heat tolerant (Jiang et al., 2020). Most of the measured traits, especially PVP, PGP and yield-related traits, exhibited high heritability (Table 2), more so under HS than normal conditions, indicating the possibility of transferring the set traits into high-yield in heat-sensitive chickpea genotypes to improve heat tolerance and sustain yield in chickpea under HS.

Correlation studies indicated damage to membranes and oxidative stress negatively affected the yield traits suggesting reduction in these traits could be linked to improved heat tolerance, which is in agreement with previous studies in lentil and chickpea (Bhandari et al., 2020), mungbean (Sharma et al., 2016), and wheat (ElBasyoni et al., 2017). Similarly, reduced lipid peroxidation, measured as MDA, might improve heat tolerance, as reported in heat tolerant genotypes of other crops such as tomato (Zhou et al., 2019) and mungbean (Sharma et al., 2016). Chlorophyll, chlorophyll fluorescence and stomatal conductance had a positive correlation with pod number and seed yield indicating these traits could be vital in determining the heat tolerance. Some previous studies also report positive correlation of chlorophyll and chlorophyll fluorescence with heat tolerance in common bean (Petkova et al., 2007) and lentil (Sita et al., 2017). Considering this, stable leaf integrity and function in terms of photosynthesis under HS would be highly vital in improving heat tolerance in chickpea.

## Conclusion

Genetic variability in various physiological, yield, and yield-related traits was assessed over 2 years in 39 chickpea genotypes under normal conditions and HS during reproductive and post-reproductive stages. A selected set of contrasting genotypes was validated further under control and HS conditions in a growth chamber. We found that genetic variability for pollen viability and pollen germination under HS could be used to select heat-tolerant chickpea genotypes, as the impact of HS on pollen germination and pollen development had a direct impact on pod set%, SSWP, NPP, and SYP. Moreover, yield and yield-related traits had positive and significant correlations with chlorophyll content, pollen viability%, pollen germination%, stomatal conductance, and PS II function under HS, indicating the potential to use these traits to improve heat tolerance in chickpea. The candidate genotypes GNG469, GNG1488, GNG1499, GNG1969, GNG469, GNG1499, PantG 186, RSG 888, and CSJ515 had high PSP, NPP, and SSWP, SYP, and low decline in pollen viability and pollen germination under HS, and could be used to transfer these traits into high-yielding heat-sensitive chickpea genotypes to increase HS tolerance.

## Data Availability Statement

The original contributions presented in the study are included in the article/Supplementary Material; further inquiries can be directed to the corresponding author/s.

## Author Contributions

PD conducted the experiment. UJ helped in analysis and writing part of the manuscript. VP contributed in providing chickpea lines. KSh, SKP, and PJP helped in analysis of data. SKP helped in writing part of the manuscript. KSi and PP helped in editing the entire manuscript. HN developed the idea of this experiment. All authors contributed to the article and approved the submitted version.

## Conflict of Interest

The authors declare that the research was conducted in the absence of any commercial or financial relationships that could be construed as a potential conflict of interest.

## Publisher’s Note

All claims expressed in this article are solely those of the authors and do not necessarily represent those of their affiliated organizations, or those of the publisher, the editors and the reviewers. Any product that may be evaluated in this article, or claim that may be made by its manufacturer, is not guaranteed or endorsed by the publisher.
