# Supplementary material for: Response of Physiological, Reproductive Function and Yield Traits in Cultivated Chickpea (Cicer arietinum L.) Under Heat Stress
Source: Front Plant Sci. 2022 May 25;13:880519. doi: 10.3389/fpls.2022.880519 (PMC9202580; doi:10.3389/fpls.2022.880519)
Supplement: Supplementary file 1 [file Data_Sheet_1.docx]

**Supplementary Table S1:** List of chickpea genotypes used in the study

| **Genotype** | **Source** | **Status** |
| --- | --- | --- |
| GNG-1581 | Sriganganagar, India | Released variety |
| GNG-1958 | Sriganganagar, India | Released variety |
| GNG-2144 | Sriganganagar, India | Released variety |
| GNG-2171 | Sriganganagar, India | Released variety |
| GNG-2207 | Sriganganagar, India | Released variety |
| GNG-2299 | Sriganganagar, India | Released variety |
| GNG-1969 | Sriganganagar, India | Released variety |
| GNG-1499 | Sriganganagar, India | Released variety |
| GNG-1488 | Sriganganagar, India | Released variety |
| GNG-469 | Sriganganagar, India | Released variety |
| GNG-663 | Sriganganagar, India | Released variety |
| RSG-888 | Durgapura, India | Released variety |
| CSJ-515 | Durgapura, India | Released variety |
| RSG-931 | Durgapura, India | Released variety |
| DCP-92-3 | Indian Institute of Pulses Research | Released variety |
| CSG-8962 | Karnal, India | Released variety |
| PUSA-547 | Indian Agricultural Research Institute Research institution, New Delhi | Released variety |
| RSG-945 | Durgapura, India | Released variety |
| KWR108 | Chandra Shekhar Azad University of Agriculture and Technology, Kanpur, India | Released variety |
| Pant G-186 | Govind Ballabh Pant University of Agriculture and Technology, Pantnagar, India | Released variety |
| IPC 014-9 | Indian Institute of Pulses Research | Advanced breeding line |
| IPC 13-8 | Indian Institute of Pulses Research | Advanced breeding line |
| IPC 14-39 | Indian Institute of Pulses Research | Advanced breeding line |
| IPC 11-29 | Indian Institute of Pulses Research | Advanced breeding line |
| IPC 11-49 | Indian Institute of Pulses Research | Advanced breeding line |
| IPC 12-59 | Indian Institute of Pulses Research | Advanced breeding line |
| ICCV 92944 | International Crops Research Institute for the Semi-arid tropics, India | Released variety |
| ICC-10685 | International Crops Research Institute for the Semi-arid tropics, India | Accession |
| RVG-202 | Shehore, India | Released variety |
| PDG-3 | Punjab Agricultural University, Ludhiana, India | Released variety |
| ICC-96030 | International Crops Research Institute for the Semi-arid tropics, India | Accession |
| ICCV-10 | International Crops Research Institute for the Semi-arid tropics, India | Released variety |
| GL 12021 | Punjab Agricultural University, Ludhiana, India | Advanced breeding line |
| GL 13001 | Punjab Agricultural University, Ludhiana, India | Released variety as PBG9 |
| GL 13042 | Punjab Agricultural University, Ludhiana, India | Released variety as PBG8 |
| GL 14001 | Punjab Agricultural University, Ludhiana, India | Advanced breeding line |
| GL 15017 | Punjab Agricultural University, Ludhiana, India | Advanced breeding line |
| GL 15026 | Punjab Agricultural University, Ludhiana, India | Advanced breeding line |
| PDG4 | Punjab Agricultural University, Ludhiana, India | Released variety |

**Supplementary Table S2:** Maximum, minimum temperature, relative humidity (RH) and light intensity during normal and late-sown (heat-stressed) the years 2017-18 and 2018-19

|  | **Season** | **Growth stage** | **Average Max/Min Temp**  **RH** |
| --- | --- | --- | --- |
| 1. | 2017–18  Normal Sown | Vegetative stage | 7.2-28.4°C (maximum) and 4.2-14.3°C (minimum)  71-87% (maximum) and 21-67% (minimum) |
|  |  | Reproductive stage | 18.3-32.4°C (maximum) and 9.2-17.3°C (minimum)  73-84% (maximum) and 17-60% (minimum) |
| 2. | 2017–18  Late-sown (heat-stressed) | Vegetative stage | 17.5°-32.5°C (maximum) and 9.4-18.4°C (minimum)  56-81% (maximum) and 19-76% (minimum) |
|  | Light intensity: 1,423–1,561 µmol m^−2^ s^−1^ | | |
|  |  | Reproductive stage | 26.4-37.7°C (maximum) and 14.2°-27.2°C (minimum)  49-83% (maximum) and 14-52% (minimum) |
| 3. | 2018-19  Normal Sown | Vegetative stage | 6.9-27.9°C (maximum) and 4.9-15.2°C (minimum)  71-87% (maximum) and 21-67% (minimum) |
|  |  | Reproductive stage | 18.3-32.4°C (maximum) and 9.2-17.3°C (minimum)  73-84% (maximum) and 17-60% (minimum) |
| 4. | 2018-19  Late-Sown  (heat-stressed) | Vegetative stage | 18.3-33.5°C (maximum) and 8.6-17.3°C (minimum)  56-81% (maximum) and 19-76% (minimum) |
|  |  | Reproductive stage | 27.3-36.4°C (maximum) and 15.1-26.4°C (minimum)  49-83% (maximum) and 14-52% (minimum) |
|  | Light intensity: 1,424–1,523 µmol m^−2^ s^−1^ | | |


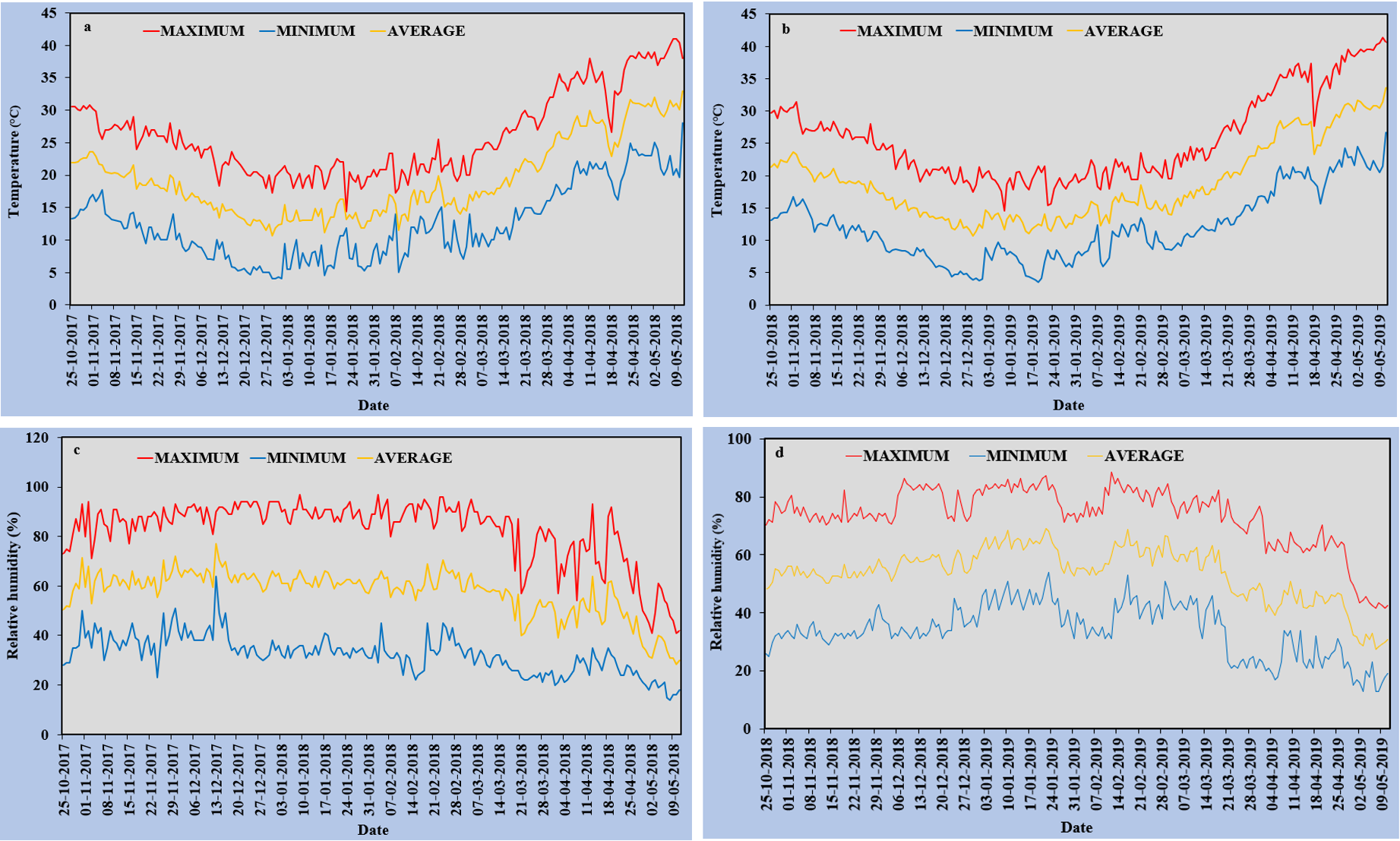


**Supplementary Fig. S1:** Weather data (maximum (max), minimum (min) and average (avg) temperature (°C), (a, b) and relative humidity RH (%) (c, d).

**Supplementary Fig. S2:** Electrolyte leakage (%) of chickpea genotypes under control normal-sown) (C) and heat stress (late-sown) (HS) environment during 2017-18 (a), 2018-19 (b) and in control environment of growth chamber (GC; C-control; HS-heat stress) (c). LSD values (P<0.05); genotype × treatment): 3.1 (2017-18), 3.4 (2018-19), 3.9 (GC). Values are means + SE. (n = 3).

**Supplementary Fig. S3:** Stomatal conductance (gs) of chickpea genotypes under control (normal-sown) (C) and heat stress (late-sown) (HS) environment during 2017-18 (a), 2018-19 (b) and in control environment of growth chamber (GC; C-control; HS-heat stress) (c). LSD values (P<0.05); genotype × treatment): 12.5 (2017-18), 15.4 (2018-19), 13.9 (GC). Values are means + SE. (n = 3).

**Supplementary Fig. S4:** SPAD chlorophyll value of chickpea genotypes under control (normal-sown) (C) and heat stress (late-sown) (HS) environment during 2017-18 (a), 2018-19 (b) and in control environment of growth chamber (GC; C-control; HS-heat stress) (c). LSD values (P<0.05); genotype × treatment): 2.6 (2017-18), 3.1 (2018-19), 2.9 (GC). Values are means + SE. (n = 3).

**Supplementary Fig. S5:** Photosystem II function (PS) of chickpea genotypes under control (normal-sown) (C) and heat stress (late-sown) (HS) environment during 2017-18 (a), 2018-19 (b) and in control environment of growth chamber (GC; C-control; HS-heat stress) (c). LSD values (P<0.05); genotype × treatment): 0.11 (2017-18), 0.13 (2018-19), 0.11 (GC). Values are means + SE. (n = 3).

**Supplementary Fig. S6:** Malondialdehyde (MDA) of chickpea genotypes under control (normal-sown) (C) and heat stress (late-sown) (HS) environment during 2017-18 (a), 2018-19 (b) and in control environment of growth chamber (GC; C-control; HS-heat stress) (c). LSD values (P<0.05); genotype × treatment): 2.4 (2017-18), 2.1(2018-19), 2.7 (GC). Values are means + SE. (n = 3).

**Supplementary Fig.S7:** Pollen viability (%) of chickpea genotypes under control (normal-sown) (C) and heat stress (late-sown) (HS) environment during 2017-18 (a), 2018-19 (b) and in control environment of growth chamber (GC; C-control; HS-heat stress) (c). LSD values (P<0.05); genotype × treatment): 10.5 (2017-18), 12.8 (2018-19), 12.1 (GC). Values are means + SE. (n = 3).

**Supplementary Fig.S8:** Pollen germination (%) of chickpea genotypes under control (normal-sown) (C) and heat stress (late-sown) (HS) environment during 2017-18 (a), 2018-19 (b) and in control environment of growth chamber (GC; C-control; HS-heat stress) (c). LSD values (P<0.05); genotype × treatment): 11.6 (2017-18), 13.5 (2018-19), 11.9 (GC). Values are means + SE. (n = 3).
